# High Immunohistochemical Expression of SETD5 as a Candidate Pathological Factor for Dedifferentiation and Prognosis in Liposarcoma

**DOI:** 10.1111/pin.70076

**Published:** 2025-12-30

**Authors:** Makoto Abe, Naoto Kubota, Ken Yamazaki, Eisuke Miura, Kaoru Hirabayashi, Masatsugu Ishii, Hirofumi Shirakawa, Kazutaka Kikuta, Yudai Murayama, Rumi Nakagawa, Hidenori Ojima

**Affiliations:** ^1^ Division of Molecular Pathology, Research Institute Tochigi Cancer Center Utsunomiya Tochigi Japan; ^2^ Department of Diagnostic Pathology Tochigi Cancer Center Utsunomiya Tochigi Japan; ^3^ Department of Musculoskeletal Oncology and Orthopedic Surgery Tochigi Cancer Center, Yonan Utsunomiya Tochigi Japan

**Keywords:** Dedifferentiated liposarcoma, Immunohistochemistry, Liposarcoma, Prognostic marker, SET Domain Containing 5 (SETD5)

## Abstract

SET domain containing 5 (SETD5), a chromatin regulator involved in adipocytic differentiation, has been identified in various cancers, but its immunohistochemical expression and prognostic significance in liposarcoma remain unclear. This study examined the immunohistochemical expression and prognostic significance of SETD5 in liposarcomas. SETD5 expression was analyzed in 100 adipocytic tumors using immunohistochemistry; these 100 tumors consisted of 24 dedifferentiated liposarcomas (DDLPS), 24 atypical lipomatous tumors/well differentiated liposarcomas (WDLPS), 12 myxoid liposarcomas, 5 pleomorphic liposarcomas, and 35 benign adipocytic tumors. SETD5 expression was assessed using the immunoreactivity score and its prognostic significance was investigated. SETD5 expression was absent in normal adipose tissue and minimal in lipomas. SETD5 expression was significantly higher in WDLPS than in lipomas (*p* = 0.01). Moreover, SETD5 expression was markedly elevated in the dedifferentiated component of DDLPS compared to the well‐differentiated component (*p* < 0.001). Pleomorphic liposarcoma showed the highest SETD5 expression levels. In DDLPS, high SETD5 expression in the dedifferentiated component correlated with worse overall survival (*p* < 0.001) but was not correlated with disease‐free survival (*p* = 0.086). Immunohistochemical expression of SETD5 significantly correlates with prognosis in DDLPS and may serve as a candidate pathological factor for dedifferentiation and prognosis.

## Graphical abstract

SET domain containing 5 (SETD5), a chromatin regulator involved in adipocytic differentiation. However, its immunohistochemical expression and prognostic significance in liposarcoma remain unclear. In our study, SETD5 expression was markedly elevated in the dedifferentiated component of dedifferentiated liposarcoma compared to the well‐differentiated component, and high SETD5 expression in the dedifferentiated component correlated with worse overall survival. SETD5 may serve as a candidate pathological factor for dedifferentiation and prognosis.

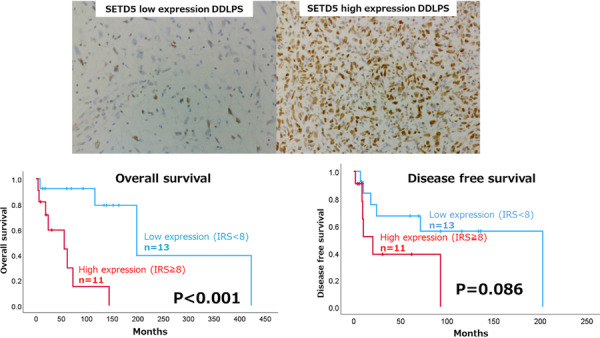

## 1 Introduction

Liposarcoma is a common type of soft tissue sarcoma, with well‐differentiated (WDLPS) and dedifferentiated (DDLPS) subtypes representing a histological continuum [1, 2]. Whereas WDLPS is characterized by mature adipocytic differentiation and an indolent clinical course, DDLPS exhibits loss of adipocytic differentiation and increased aggressiveness, often leading to poor clinical outcomes [1, 3]. Accurate identification of dedifferentiated components and precise assessment of their malignant potential are necessary for appropriate treatment strategies in liposarcoma.

SET domain containing 5 (SETD5) is a member of the histone lysine methyltransferase family; it plays a pivotal role in transcription regulation, euchromatin formation, and RNA elongation and splicing through the methylation of histone H3 on lysine 36 (H3K36) [4, 5]. In lipid metabolism, SETD5 forms a complex with the nuclear receptor corepressor (NCoR) and histone deacetylase 3 (HDAC3), acting as a co‐repressor to maintain enhancers in a hypoacetylated “primed” state [6, 7, 8]. During early adipogenesis, this complex prevents histone acetylation of enhancers for key adipogenic regulatory genes such as Cebpa and Pparg [9]. The degradation of SETD5 from the NCoR–HDAC3 complex allows for enhancer hyperacetylation, facilitating the transition to an active state and promoting adipocyte differentiation [8]. Despite these insights, the role of SETD5 in mesenchymal tumors, particularly adipocytic tumors, remains unclear. Because of the role of SETD5 in transcriptional regulation and adipocytic differentiation, its expression status may contribute to the dedifferentiated state and aggressive tumor behavior of DDLPS.

This study investigated the immunohistochemical expression of SETD5 in adipocytic tumors and its potential role as a prognostic marker in DDLPS. By analyzing SETD5 expression levels in a comprehensive cohort of adipocytic tumors, we sought to elucidate its relationship with adipocytic differentiation and clinical outcomes.

## 2 Materials and Methods

### 2.1 Case Selection

A review of the institutional pathology database at the Tochigi Cancer Center in Japan was performed for cases of adipocytic tumors diagnosed between January 2004 and December 2024. A total of 649 cases were identified: these consisted of lipomas (270 cases), spindle cell lipomas (50 cases), intramuscular lipomas (48 cases), angiolipomas (15 cases), DDLPS (54 cases), atypical lipomatous tumor/well‐differentiated liposarcomas (ALT/WDLPS, 163 cases), myxoid liposarcomas (41 cases), and pleomorphic liposarcomas (8 cases). The inclusion criteria for the current study were the availability of surgically resected specimens, traceable clinical follow‐up data, and access to paraffin‐embedded tissue blocks. Moreover, patients who died within 3 months after surgery and those with multiple primary malignancies were excluded from the study. Of the 54 cases of DDLPS, 24 were selected according to these criteria. These 24 DDLPS cases involved 19 men and 5 women and had a median follow‐up period of 82 months (maximum of 423 months).

To ensure a balanced comparison with DDLPS, 24 ALT/WDLPS (19 ALT and 5 WDLPS cases) were selected using the same inclusion criteria. Similarly, 12 myxoid liposarcomas and 5 pleomorphic liposarcomas were included following the same selection process.

For benign adipocytic tumors, representative cases considered to be typical of each entity were selected to meet the necessary sample size requirements: 20 lipomas, 5 spindle cell lipomas, 5 intramuscular lipomas, and 5 angiolipomas were selected.

A total of 100 adipocytic tumor cases were reviewed and diagnostically confirmed by two experienced pathologists (MA and NK) in accordance with the 5th edition of the WHO Classification of Tumors of Soft Tissue and Bone [1, 10, 11]. For DDLPS cases, representative sections were selected from the non‐lipogenic area, specifically the region with the highest degree of cellular atypia and the greatest number of mitotic figures.

### 2.2 Immunohistochemistry

Immunohistochemical staining for SETD5 was performed on formalin‐fixed, paraffin‐embedded tumor sections. Tissue sections (4 μm thick) were deparaffinized with xylene and rehydrated using graded ethanol solutions. Endogenous peroxidase activity was quenched using a 0.3% hydrogen peroxide solution for 30 min. Heat‐induced antigen retrieval was performed in citrate buffer (pH 6.0) at 121°C for 10 min. Sections were blocked with 2.5% horse serum before incubation with a rabbit polyclonal anti‐SETD5 antibody (clone HPA035574, Sigma Aldrich, St. Louis, USA) at a 1:400 dilution for 1 h at room temperature. The Dako REAL EnVision‐HRP system (DAKO, Glostrup, Denmark) was used for detection, and 3,3′‐diaminobenzidine chromogen was applied for visualization. Slides were counterstained with hematoxylin, dehydrated, and mounted. Internal controls included endothelial cells and lymphocytes, which consistently express SETD5.

### 2.3 Scoring of Immunohistochemical Staining

SETD5 immunoreactivity was semi‐quantitatively assessed using the immunoreactive score (IRS) method [12]. The IRS incorporates two parameters:

#### 2.3.1 Proportion of Positively Stained Neoplastic Cells

- 0: No stained cells
- 1: < 10% of cells stained
- 2: 10–50% of cells stained
- 3: 51–80% of cells stained
- 4: > 80% of cells stained

#### 2.3.2 Staining Intensity

- 0: No staining
- 1: Weak staining
- 2: Moderate staining
- 3: Strong staining

**Figure 1:**
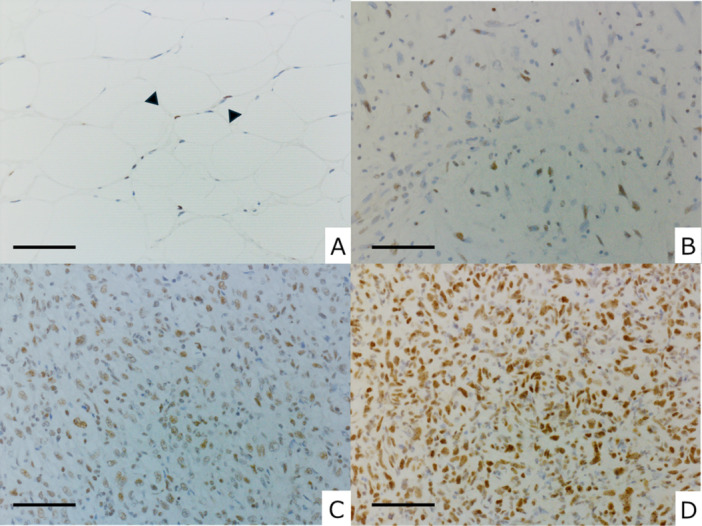
Representative immunoreactive score images for assessment of SETD5 expression. Negative immunostaining of SETD5 was evident in normal adipose tissue (A). Minimal expression was observed in background capillaries (arrowheads). Shown are cases of dedifferentiated liposarcoma with immunoreactivity scores of 4, 6, and 12 (B, C, and D, respectively). SETD5 was moderately expressed in approximately 30% (B) and 60% (C) of tumor cells with a score of 4 or 6 and strongly expressed in approximately 90% (D) of tumor cells with a score of 12. Scale bars: 100 μm (A), 50 μm (B, C, and D).

The IRS score was calculated as the product of these two parameters, yielding a total score ranging from 0 to 12 for each case. Staining intensity was judged relative to the positivity of vascular endothelial cells: staining stronger than endothelial cells was classified as strong, comparable staining as moderate, and weaker staining as weak. Cases with an IRS ≥ 8 were assigned to the high expression group, whereas cases with an IRS < 8 were assigned to the low expression group [12, 13, 14, 15, 16]. Representative images of IRS evaluation are shown in Figure 1A–D.

Tissue sections were examined by an experienced pathologist (MA and NK) who was blinded to clinical outcomes. Immunohistochemical staining was assessed independently by both pathologists, and discrepancies in IRS were resolved through a joint review. In DDLPS, IRS was measured separately for the dedifferentiated and well‐differentiated components to evaluate differences in SETD5 expression between these regions. For comparison, IRS was also assessed in normal adipose tissue located at a sufficient distance from the tumor.

### 2.4 The Pancancer Atlas Cohort and Survival Analysis

RNA‐seq and clinical data for sarcoma cases were selected from The Cancer Genome Atlas (TCGA), PanCancer Atlas cohort, via cBioPortal [17, 18]. From 255 sarcoma cases with available RNA‐seq data, two cases of desmoid fibromatosis were excluded. The remaining cohort comprised 59 dedifferentiated liposarcoma cases and 194 non‐adipocytic sarcoma cases: the latter included leiomyosarcoma (*n* = 100), undifferentiated pleomorphic sarcoma (*n* = 50), myxofibrosarcoma (*n* = 25), synovial sarcoma (*n* = 10), and malignant peripheral nerve sheath tumor (*n* = 9). SETD5 mRNA expression values were stratified by quartile. The top 25% was defined as the high‐expression group and the bottom 25% as the low‐expression group. Overall survival and disease‐free survival were estimated using the Kaplan–Meier method and compared using the log‐rank test.

### 2.5 Statistical Analysis

Statistical analyses were performed using SPSS software version 29.0.2.0 (Armonk, New York, USA). Associations between SETD5 expression and clinicopathological features were assessed using the Chi‐square test, Fisher′s exact test, or Mann‐Whitney U test, as appropriate. Kaplan–Meier survival analysis was employed to evaluate the prognostic significance of SETD5 expression, with the log‐rank test used to compare survival distributions. For DDLPS, SETD5 expression in the dedifferentiated component was used as the representative value for Kaplan–Meier survival analysis. Early postoperative recurrences and deaths (within 3 months) were excluded from the disease‐free survival analysis. A P‐value of less than 0.05 was considered statistically significant.

## 3 Results

### 3.1 SETD5 Expression in Atypical Lipomatous Tumor/Wdlps and DDLPS

**Figure 2:**
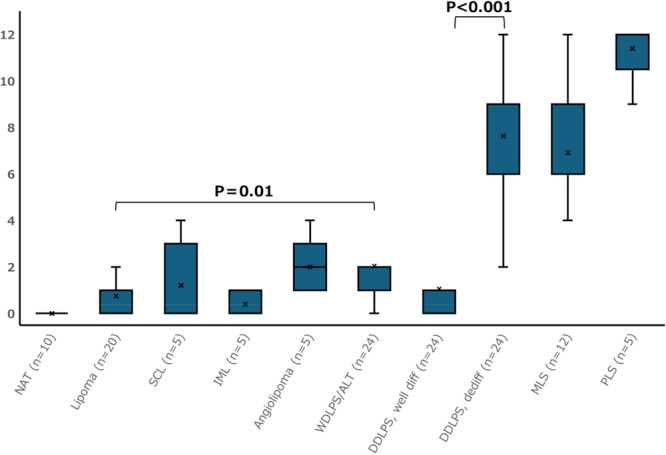
Correlations between the SETD5 immunoreactivity score (IRS) and the different types of adipocytic tumors. IRS was low in benign adipocytic tumors and well‐differentiated liposarcomas (WDLPS). But the Mann‐Whitney U test indicated that there were significant differences in expression between lipomas *(p* = 0.01*)* and WDLPS and between the well‐differentiated and dedifferentiated components of dedifferentiated liposarcoma (DDLPS) (*p* < 0.001). NAT, normal adipose tissue; SCL, spindle cell lipoma; IML, intramuscular lipoma; DDLSP, well diff, well differentiated component of DDLPS; dediff, dedifferentiated component of DDLPS; MLS, myxoid liposarcoma; PLS, pleomorphic liposarcoma. Box plots show the interquartile range (IQR; Q1–Q3), with central lines indicating the median and whiskers extending to 1.5×IQR. Cross marks (×) represent the mean. P‐values are shown only for variables with pathological importance and were calculated using the Mann‐Whitney U test.

**Figure 3:**
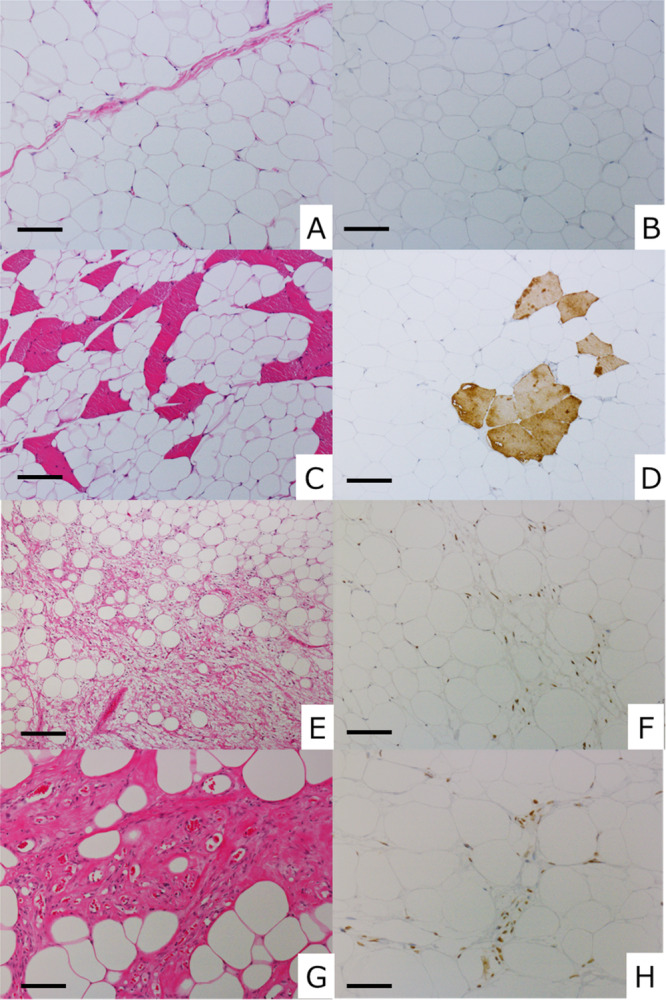
Representative histological findings of SETD5 expression in benign adipocytic tumors. In lipoma (A, B) and intramuscular lipoma (C, D), positive immunohistochemical findings of SETD5 were rarely observed in adipose tissue, whereas strong positive findings were observed in muscle tissue and acted as a positive internal control. However, in spindle cell lipoma (E, F) and angiolipoma (G, H), positive immunohistochemical findings were often observed in the spindle‐shaped adipose tissue and in capillaries and fibrous tissue (A, C, E, G: H&E staining; B, D, F, H: SETD5 staining). Scale bars: 200 μm (A–F), 100 μm (G and H).

The SETD5 IRS in adipocytic tumors are summarized in Figure 2. SETD5 expression was absent in normal adipose tissue, whereas the median IRS was low in lipoma (0.8) and intramuscular lipoma (0.4) and slightly higher in spindle cell lipoma (1.2) and angiolipoma (2.0). The distribution of SETD5 expression in these adipocytic tumors is shown in Figure 3.

**Figure 4:**
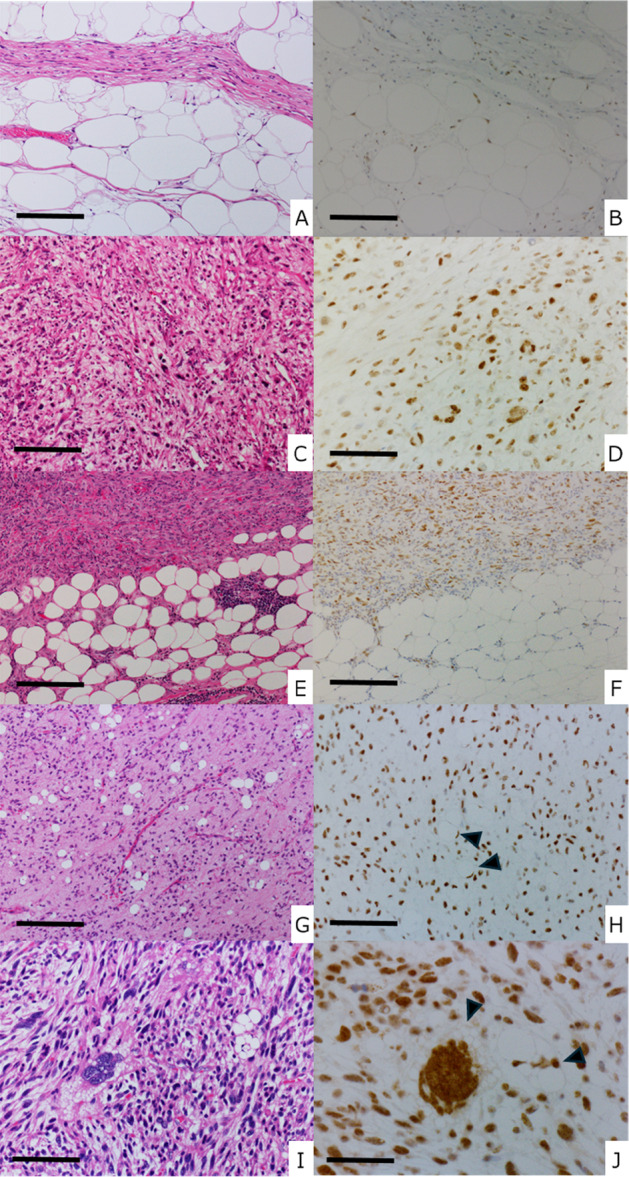
Representative histological findings of SETD5 expression in liposarcomas. In well‐differentiated liposarcoma (WDLPS) (A), weak immunohistochemical expression of SETD5 was observed in some atypical adipocytes and stromal cells within the fibrous septa (B). In dedifferentiated liposarcoma (DDLPS) (C), strong nuclear positivity was observed in spindle‐shaped and pleomorphic tumor cells (D). In cases of WDLPS (lower portion) and DDLPS (upper portion) components within the same tumor (E), positive immunostaining of SETD5 was observed at the WDLPS–DDLPS boundary (F). In the WDLPS component, weak and partial SETD5 expression was observed, whereas strong nuclear positivity was seen in the DDLPS component (F). In myxoid liposarcoma (G), strong nuclear positivity was observed in both spindle‐shaped tumor cells and signet‐ring‐like atypical adipocytes (H, arrowheads). In pleomorphic liposarcoma (I), diffuse strong nuclear positivity was seen (J). Pleomorphic lipoblasts with multiple cytoplasmic lipid droplets also exhibit strong SETD5 nuclear staining (J, arrowheads). (A, C, E, G, I: H&E staining; B, D, F, H, J: SETD5 staining). Scale bars: 100 μm (A–D, and I), 400 μm (E and F), 200 μm (G and H), 50 μm (J).

In ALT/WDLPS, SETD5 expression was predominantly observed in neoplastic stromal cells within the fibrous septa, whereas mature adipocytic tumor cells exhibited weak nuclear expression of SETD5 (Figure 4A and B). Both neoplastic adipocytes and stromal cells exhibited significantly higher SETD5 expression than lipomas (Figure 2, p = 0.01).

In DDLPS, the dedifferentiated components demonstrated significantly stronger and diffuse SETD5 expression (mean IRS, 7.6) compared with the well‐differentiated components (mean IRS, 1.1), highlighting a strong correlation between dedifferentiation and increased SETD5 expression (Figure 4C–F). Statistical analysis confirmed that SETD5 expression was significantly higher in the dedifferentiated component than in the well‐differentiated component (*p* < 0.001).

### 3.2 SETD5 Expression in Other Liposarcomas

In myxoid liposarcomas, SETD5 expression was observed in both short spindle‐shaped tumor cells and signet‐ring‐like tumor cells, with strong nuclear positivity detected in these distinct cell types with a mean IRS of 6.9 (Figure 4G and H). In pleomorphic liposarcomas, nuclear SETD5 expression was present in both mononuclear and multinucleated tumor cells with a mean IRS of 11.4 (Figure 4I and J), highlighting the broad expression profile of SETD5 across different tumor cell morphologies.

### 3.3 Clinicopathological Features and SETD5 Expression in Liposarcomas

A comprehensive summary of the clinicopathological characteristics of liposarcomas including WDLPS, DDLPS, myxoid liposarcoma, and pleomorphic liposarcoma, are summarized in Table 1. Analysis of SETD5 expression revealed that tumors with high SETD5 expression exhibited significantly higher mitotic activity (*p* < 0.001) and increased vascular invasion (*p* < 0.001). Histologically, all five cases of WDLPS were classified as SETD5 low expression, whereas all five cases of pleomorphic liposarcoma were classified as SETD5 high expression. In contrast, DDLPS and myxoid liposarcoma showed a balanced distribution of low and high SETD5 expression.

**Table 1:** Clinicopathological comparison of SETD5 expression across liposarcoma subtypes.

Characteristic	All cases [median (range) or n (%)]	SETD5 low expression [median (range) or n (%)]	SETD5 high expression [median (range) or n (%)]	P‐value
Total	46	25	21	
Sex (M:F)	31:15	15:10	16:5	0.19^a^
Age at diagnosis	67.5 (24.0–88.0)	72.0 (24.0–84.0)	61.0 (33.0–88.0)	0.38^b^
Tumor size (cm)	14.0 (2.5–37.0)	13.0 (5.0–37.0)	15.0 (2.5–30.0)	0.65^b^
Site (intraabdominal)	13 (28.9%)	6 (24%)	7 (33.3%)	0.35^a^
Mitosis (per 10HFP)	8.0 (0.0–92.0)	5.0 (0.0–28.0)	15.0 (2.0–92.0)	**< 0.001** b
Vascular invasion	15/46 (32.6%)	4/25 (16%)	11/21 (52.4%)	**< 0.01** a
Surgical margin positive	24/46 (52.2%)	12/25 (48%)	12/21 (57.1%)	0.43^a^
FNCLCC grade				
Grade 1	5/46 (10.9%)	5/25 (20%)	0/21 (0%)	**< 0.05** a
Grade 2	23/46 (50%)	13/25 (52%)	10/21 (47.6%)	0.5^a^
Grade 3	18/46 (39.1%)	7/25 (28%)	11/21 (52.4%)	0.08^a^
Histology				
WDLPS	5	5 (100%)	0 (0%)	**0.031** c
DDLPS	24	13 (54.7%)	11 (45.3%)	0.42^c^
Myxoid liposarcoma	12	8 (66.7%)	4 (33.3%)	0.19^c^
Pleomorphic liposarcoma	5	0 (0%)	5 (100%)	**0.031** c

Cases with IRS ≥ 8 were classified as having high expression, whereas cases with IRS < 8 were classified as low expression.

Of the 24 WDLPS/ALT cases, only 5 WDLPS cases were included in the analysis.

ALT, atypical lipomatous tumor; DDLPS, dedifferentiated liposarcoma; HPF, high‐power field; WDLPS, well‐differentiated liposarcoma.

^a^ Chi‐square Test,

^b^ Mann‐Whitney U test,

^c^ Binomial test.

Table 2 shows a comparison of the dedifferentiated components of DDLPS in the high and low SETD5 expression groups. The proportion of men was significantly higher in the SETD5 high expression group (*p* < 0.001). Although mitotic activity and vascular invasion showed a trend toward higher levels in the high SETD5 expression group, these differences did not reach statistical significance (*p* = 0.15 and *p* = 0.12, respectively).

**Table 2:** Clinicopathological comparison of SETD5 expression in dedifferentiated liposarcoma.

Characteristic	All cases [median (range) or *n* %]	SETD5 low expression [median (range) or *n* %]	SETD5 high expression [median (range)] or *n* %]	*P*‐value
Total	24	13	11	
Sex (M:F)	19:5	9:4	10:1	**< 0.001** a
Age at diagnosis (years)	71 (45–88)	75 (66–84)	65.0 (45.0–88.0)	0.072^b^
Tumor size (cm)	15.0 (8.0–30.0)	14.5 (8.0–26.0)	15.5 (9.5–30.0)	0.65^b^
Site (intraabdominal)	12/24 (50%)	5/13 (46%)	6/11 (54%)	0.35^a^
Mitosis (per 10HPF)	13 (3–46)	11 (3–28)	20 (6–46)	0.15^b^
Vascular invasion	9/24 (37.5%)	3/13 (23%)	6/11 (55%)	0.12^a^
Surgical margin positive	18/24 (75%)	9/13 (69%)	9/11 (81%)	0.41^a^

HPF, high‐power field.

^a^ Fisher′s exact test,

^b^ Mann‐Whitney U test.

### 3.4 Prognostic Analysis of SETD5 Expression in DDLPS

**Figure 5:**
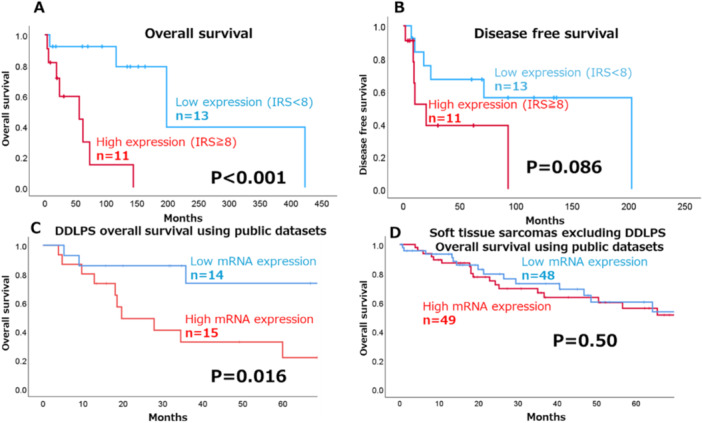
Prognostic analysis stratified by the level of SETD5 expression in dedifferentiated liposarcoma (DDLPS). Kaplan–Meier analysis revealed that (A) overall survival (OS) was significantly shorter in the high SETD5 expression group than in the low expression group (*p* < 0.001); however, (B) for disease‐free survival (DFS), there was no significant difference between the high and low expression groups. (C) Kaplan–Meier analysis of 59 cases of DDLPS from the TCGA cohort revealed that patients with high SETD5 mRNA expression (upper quartile, 15 cases) had significantly shorter OS than those with low SETD5 mRNA expression (lower quartile, 14 cases) (*p* = 0.016). (D) Kaplan–Meier analysis of 194 non‐adipocytic sarcoma cases in the TCGA cohort (including leiomyosarcomas, undifferentiated pleomorphic sarcomas, myxofibrosarcomas, synovial sarcomas, and malignant peripheral nerve sheath tumors) showed no significant difference in OS between the high and low SETD5 expression groups, defined by the upper and lower quartiles, (49 and 48 cases, respectively; *p* = 0.50).

In our cohort of 24 DDLPS cases, high SETD5 expression was significantly associated with worse overall survival (OS) (*p* < 0.001, Figure 5A). In contrast, disease‐free survival (DFS) showed no significant difference between the low and high SETD5 expression groups (*p* = 0.086, Figure 5B). In the TCGA cohort, the 59 DDLPS cases with high SETD5 mRNA expression (upper quartile, 15 cases) had significantly shorter OS than those with low SETD5 mRNA expression (lower quartile, 14 cases) (*p* = 0.016, Figure 5C); this result further supports the prognostic relevance in DDLPS patients of SETD5 at the transcriptomic level. In the same TCGA cohort, DFS was not significantly different between the high and low expression groups (*p* = 0.56, data not shown). Furthermore, in the TCGA sarcoma cohort [194 cases of non‐adipocytic sarcoma: leiomyosarcoma (*n* = 100), undifferentiated pleomorphic sarcoma (*n* = 50), myxofibrosarcoma (*n* = 25), synovial sarcoma (*n* = 10), and malignant peripheral nerve sheath tumor (*n* = 9)] no significant differences in OS were observed between the SETD5 high and low mRNA expression groups (*p* = 0.50, Figure 5D), suggesting that the prognostic impact of SETD5 may be specific to adipocytic sarcomas.

## 4 Discussion

This study provides the first comprehensive evaluation of SETD5 immunohistochemical expression across a spectrum of adipocytic tumors and demonstrates an inverse correlation between SETD5 expression and adipocytic differentiation. Benign adipocytic tumors, such as lipomas and intramuscular lipomas, showed little to no SETD5 expression, consistent with their well‐differentiated maturation. In contrast, liposarcomas, particularly DDLPS, myxoid liposarcoma, and pleomorphic liposarcomas, exhibited significantly higher SETD5 immunohistochemical expression. In DDLPS and pleomorphic liposarcoma, higher IRSs were observed in areas with greater nuclear pleomorphism and cellularity, whereas in myxoid liposarcoma, SETD5 was detected not only in round‐cell components but also in spindle tumor cells with minimal atypia (Figure 4H). This finding emphasized the strong association between SETD5 expression and decreased adipocytic differentiation or dedifferentiation, further highlighting the potential role of SETD5 in tumor progression.

In the current study, SETD5 immunohistochemical expression was particularly pronounced in the dedifferentiated components of DDLPS compared with the well‐differentiated parts, suggesting the potential of SETD5 as a marker identifying dedifferentiation. While dedifferentiation is often readily recognizable as a distinct undifferentiated pleomorphic sarcoma component, some tumors exhibit only mild atypia or a gradual transition, making diagnosis challenging [2, 16, 19]. In such cases, SETD5 immunohistochemical expression may support the identification of dedifferentiation. Furthermore, as shown in Table 2, high SETD5 expression in the dedifferentiated component of DDLPS was associated with increased mitotic activity, a higher prevalence of vascular invasion, observed in 55% of cases compared with 23% in the low expression group Although these differences did not reach statistical significance in our cohort, SETD5 may contribute to the stratification of DDLPS, highlighting its potential clinical utility. Interestingly, although the current study did not quantitatively assess the proportion of the dedifferentiated components in DDLPS, it is notable that regardless of their extent, SETD5 immunohistochemical expression within the dedifferentiated components of DDLPS was significantly associated with worse OS. These findings suggest that SETD5 expression may serve as a prognostic marker independent of the quantity of dedifferentiation, emphasizing its role in tumor aggressiveness.

Recent studies have identified SETD5 as a prognostic marker in various human cancers, in which its overexpression is linked to tumor progression and poor clinical outcomes. In breast cancer, SETD5 enhances tumor growth by regulating glycolysis in cancer stem‐like cells, thereby promoting disease progression and metastasis [19, 20]. Similarly, in non‐small‐cell lung cancer, SETD5 overexpression correlates with advanced tumor stages and lymph node metastasis. In colorectal cancer, SETD5 knockdown suppresses tumor cell growth and stemness via the PI3K/AKT/mTOR pathway, suggesting the involvement of SETD5 in tumorigenesis [21, 22]. Supporting these prior findings, our study demonstrated a similar trend in liposarcoma, particularly in DDLPS, where high SETD5 expression was associated with worse prognosis. Kaplan–Meier survival analysis revealed that DDLPS patients with high SETD5 expression had significantly shorter OS (*p* < 0.001), and although the difference in DFS was not statistically significant, a trend toward worse DFS was observed; these findings reinforce the potential role of SETD5 as an adverse prognostic marker.

Adipocytic differentiation markers, such as adipophilin, perilipin, and peroxisome proliferator activated receptor gamma, are well‐established markers of adipocytic differentiation, but their expression may be lost in high‐grade dedifferentiated components [23, 24, 25]. In contrast, SETD5 demonstrated a distinct expression pattern, being prominently expressed in poorly differentiated and dedifferentiated tumor components where lipid droplets are absent. Interestingly, high SETD5 expression was also observed in some lipid‐containing tumor cells, such as signet‐ring cells in myxoid liposarcomas and pleomorphic lipoblasts in pleomorphic liposarcomas (Figure 4H and J, arrowheads). Despite the presence of cytoplasmic lipid droplets, these cells exhibited SETD5 expression, suggesting that lipid accumulation in these cells may not reflect normal adipocytic metabolism but rather tumor‐associated aberrant lipid metabolism [26].

This study has several inherent limitations because of the nature of the disease and the cohort size. The first limitation is related to the sample size and the rarity of some tumor types. Because liposarcomas and other relevant sarcomas are rare tumors, the overall number of cases in our cohort was relatively small; this scarcity restricted the scope of the investigation exclusively to adipocytic tumors and limited the assessment primarily to univariate analysis. The small number of available cases also meant that we were unable to include atypical spindle cell/pleomorphic lipomatous tumors, a recently recognized entity added to the fifth edition of the WHO Classification of Tumors of Soft Tissue and Bone. Furthermore, due to sample size constraints and the ambiguity in classifying the histological subtypes of ALT/WDLPS (lipoma‐like, inflammatory, and sclerosing), we did not stratify SETD5 expression based on these subtypes. Although we did not perform a subtype‐based analysis, we quantified for each case the ratio (range 0 to 1.0) of neoplastic adipose tissue to tumor area on sections and examined its correlation with SETD5 immunoreactivity score using Spearman′s rank correlation. We plotted these values to construct Supporting Information S1 Figure 1. This analysis showed a negative correlation (Spearman ρ = −0.79, *p* < 0.001, *n* = 67, Supporting Information S1 Figure 1) and suggested that tumors with more non‐adipocytic components tend to exhibit higher SETD5 scores. These findings imply that tumors with few neoplastic adipocytes, corresponding to the sclerosing variant of ALT/WDLPS, show particularly high SETD5 expression and suggest a possible association with dedifferentiation. To verify the clinical significance of our findings, further investigation will be necessary with larger cohorts, including non‐adipocytic sarcomas.

The second limitation concerns potential cohort imbalance. With a male‐to‐female ratio of 19:5 in DDLPS cases, our cohort was somewhat imbalanced. Typically, DDLPS occurs equally in men and women¹, and this imbalance may have influenced the analysis of OS and DFS. However, it is noteworthy that an analysis of 59 DDLPS cases from the TCGA cohort via cBioPortal showed no significant differences in OS or DFS between men and women, suggesting that the impact of the male:female imbalance in the current cohort is likely to be limited [17, 18, 27] (data not shown).

In conclusion, this study demonstrates that SETD5 is a useful immunohistochemical marker of dedifferentiation in liposarcoma and a prognostic biomarker in DDLPS. High SETD5 expression in the dedifferentiated component of DDLPS correlated with worse OS, suggesting its potential for prognostic stratification within this subtype. The inverse relationship between SETD5 expression levels and adipocytic differentiation further supports the role of SETD5 in tumor progression and aggressiveness. These findings indicate that SETD5 expression may support the diagnosis of dedifferentiation and serve as a prognostic indicator, thereby contributing to risk assessment in DDLPS.

## Author Contributions

Makoto Abe (MA) and Hidenori Ojima contributed significantly to the study′s conception, design, data acquisition, and interpretation. MA and Naoto Kubota evaluated and reviewed the histopathological diagnosis and immunohistochemical staining. Ken Yamazaki, Eisuke Miura, Kaoru Hirabayashi, Masatsugu Ishii and Hirofumi Shirakawa provided expertise in histopathology, guidance on experimental techniques, and data interpretation. Kazutaka Kikuta, Yudai Murayama and Rumi Nakagawa collected clinical data and performed the surgery. MA drafted the initial manuscript, which was critically revised and approved by all authors.

## Ethical Statement

This study was conducted in accordance with the ethical standards of the Declaration of Helsinki and was approved by the Institutional Review Board of the Tochigi Cancer Center (number 24‐A002). Patient data were handled with the utmost care to ensure confidentiality and anonymity. All personal identifiers were removed, and data were processed in a manner that prevents individual identification. The ethical safeguards employed ensure compliance with institutional and international standards for research involving human subjects.

## Consent

In accordance with institutional policy, patient consent for participation was obtained through an opt‐out approach. Information regarding the study was made publicly available, allowing patients the opportunity to decline inclusion.

## Conflicts of Interest

The authors declare no conflicts of interest.

## Supporting information

Supporting Information S1 data 20251123.

Supporting Information S1 Figure 1 20251128 final.

## Data Availability

All data generated or analyzed during this study are included in this published article. Further inquiries can be directed to the corresponding author.
