# Supplementary material for: High Immunohistochemical Expression of SETD5 as a Candidate Pathological Factor for Dedifferentiation and Prognosis in Liposarcoma
Source: Pathol Int. 2025 Dec 30;76(1):e70076. doi: 10.1111/pin.70076 (PMC12835964; doi:10.1111/pin.70076)
Supplement: Supplementary file 2 — Supporting Information S1 Figure 1 20251128 final. [file PIN-76-0-s002.docx]

**Supplementary figure 1.**


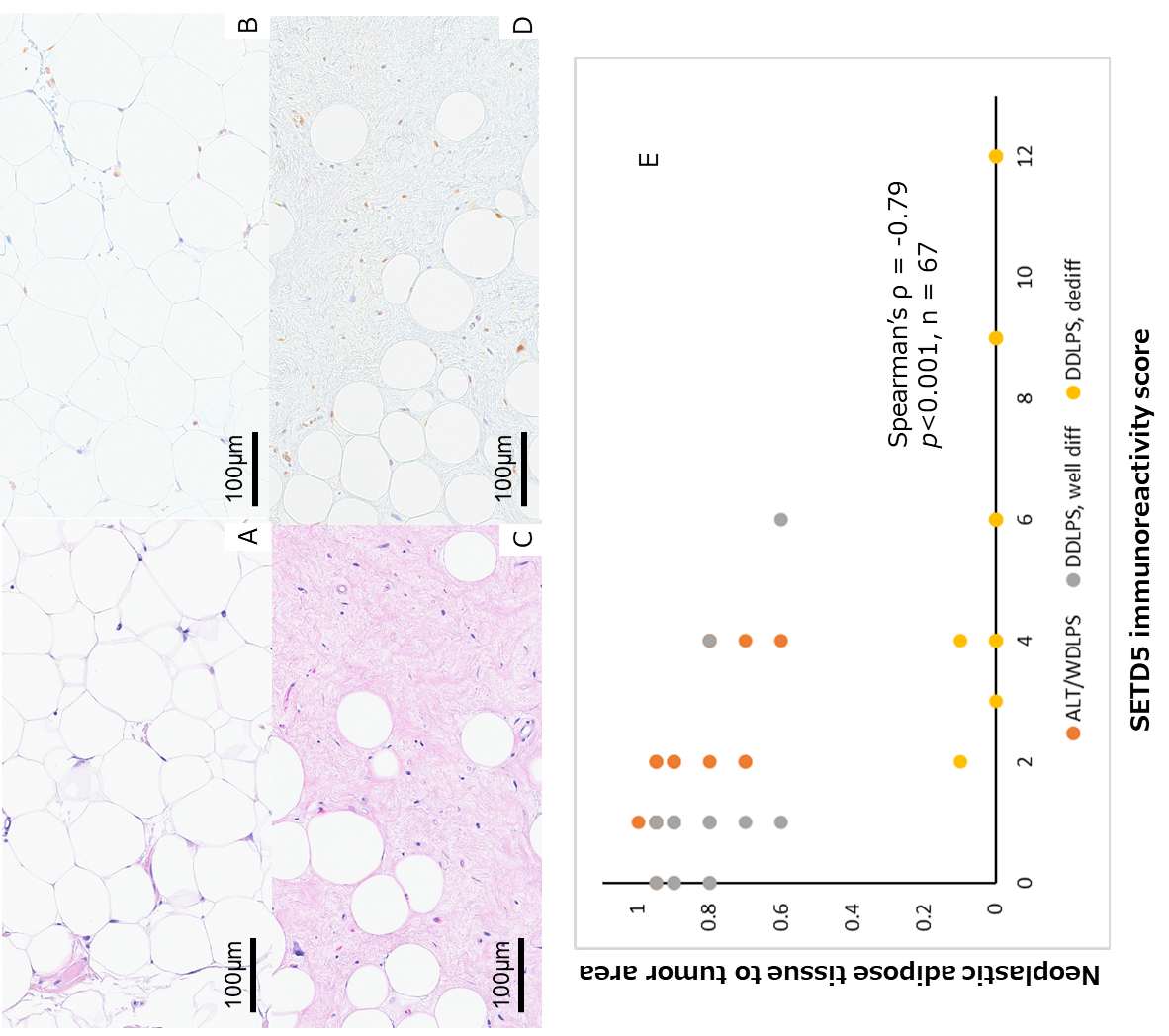


**Supp. Fig. 1:** Representative cases of atypical lipomatous tumor/well-differentiated liposarcoma (ALT/WDLPS) with different levels of adipocytic content and SETD5 expression, i.e., adipocyte-rich ALT/WDLPS with minimal SETD5 expression (A, B) and adipocyte-poor, sclerosing-type ALT/WDLPS with strong SETD5 positivity (C, D). In an ALT/WDLPS composed almost entirely of neoplastic adipocytes (A), SETD5 expression was detected only in a very small subset of neoplastic adipocytes (B). In contrast, an ALT/WDLPS with non-adipocytic area corresponding to the sclerosing subtype (C) showed more SETD5 positivity in stromal tumor cells (D). Using the dataset provided as Supporting Information S1 data, the scatter plot (E) showed a strong negative correlation (Spearman ρ = −0.79, *p*<0.001, n = 67) between the ratio of neoplastic adipocytes to tumor area on the section and the SETD5 immunoreactivity score. (A, C) H&E staining, (B, D) SETD5 staining.
